# Regional Brain Analysis of Modified Amino Acids and Dipeptides during the Sleep/Wake Cycle

**DOI:** 10.3390/metabo12010021

**Published:** 2021-12-27

**Authors:** Theodosia Vallianatou, Nicholas B. Bèchet, Mario S. P. Correia, Iben Lundgaard, Daniel Globisch

**Affiliations:** 1 Science for Life Laboratory, Department of Chemistry-BMC, Uppsala University, Box 599, SE-75124 Uppsala, Sweden; theodosia.vallianatou@kemi.uu.se (T.V.); Mario.correia@kemi.uu.se (M.S.P.C.); 2 Department of Experimental Medical Science, Lund University, SE-22362 Lund, Sweden; nicholas.bechet@med.lu.se (N.B.B.); iben.lundgaard@med.lu.se (I.L.); 3 Wallenberg Centre for Molecular Medicine, Lund University, SE-22362 Lund, Sweden

**Keywords:** dipeptides, amino acids, modifications, sleep/wake cycle, brain regions, metabolomics, mass spectrometry

## Abstract

Sleep is a state in which important restorative and anabolic processes occur. Understanding changes of these metabolic processes during the circadian rhythm in the brain is crucial to elucidate neurophysiological mechanisms important for sleep function. Investigation of amino acid modifications and dipeptides has recently emerged as a valuable approach in the metabolic profiling of the central nervous system. Nonetheless, very little is known about the effects of sleep on the brain levels of amino acid analogues. In the present study, we examined brain regional sleep-induced alterations selective for modified amino acids and dipeptides using Ultra-high performance liquid chromatography-MS/MS (UHPLC-MS/MS) based metabolomics. Our approach enabled the detection and identification of numerous amino acid-containing metabolites in the cortex, the hippocampus, the midbrain, and the cerebellum. In particular, analogues of the aromatic amino acids phenylalanine, tyrosine and tryptophan were significantly altered during sleep in the investigated brain regions. Cortical levels of medium and long chain *N*-acyl glycines were higher during sleep. Regional specific changes were also detected, especially related to tyrosine analogues in the hippocampus and the cerebellum. Our findings demonstrate a strong correlation between circadian rhythms and amino acid metabolism specific for different brain regions that provide previously unknown insights in brain metabolism.

## 1. Introduction

Sleep is a vital biological function for the survival of numerous species, and circadian rhythms play a central role in homeostatic mechanisms [1]. The brain is an important tissue affected by the sleep/wake cycle, and understanding the underlying neurochemical and metabolic processes that regulate the brain during sleep is of high importance. Sleep has been associated with increased brain clearance of neurotoxic metabolites and altered energetics compared to wakefulness [2,3,4]. Several metabolic pathways, such as purine and glucose/lactate metabolism, have been linked to circadian alterations in brain function, while an emerging number of metabolomics studies focus on unraveling novel sleep-specific metabolites [5].

Amino acid metabolism has a key function in the central nervous system (CNS). Amino acids can regulate neuronal activity via multiple mechanisms, ranging from the synthesis of neurotransmitters, e.g., the precursor amino acids phenylalanine, tyrosine and tryptophan, to direct neurotransmission and neuromodulation, e.g., the excitatory neurotransmitters glutamate and aspartate as well as the inhibitory neurotransmitter glycine [6]. Alterations in amino acid metabolism in the brain have been reported during sleep [7]. However, little is known regarding the circadian functionality of amino acid analogues, such as modified amino acids and dipeptides. These analogues, demonstrating different physicochemical and biological properties compared to single amino acids, are yet to be understood [8,9]. Nevertheless, an increasing interest in their involvement in pathophysiological conditions and their role as potential disease biomarkers has recently been demonstrated in rodent models as well as in human samples [10,11,12,13,14,15,16,17,18,19,20,21]. *N*-Acylated amino acids are a large group of bioactive molecules involved in many physiological functions, such as molecular signaling, energy homeostasis and oxidative metabolism [14,22,23,24,25]. *N*-Acylglycine, which is widely distributed in the central nervous system (CNS), was described to have several neuro-physiological properties, such as analgesic and anti-inflammatory activity [14,26]. A wide range of dipeptides, endogenously synthesized but also administered, have shown multiple neuroactive effects including anxiolytic, anti-depressant activities and even memory enhancement [12,13,18]. Dipeptide alterations have also been linked to neurodegenerative conditions, such as Alzheimer’s disease [11]. One of the most investigated neuroactive dipeptides is carnosine (beta-alanyl-L-histidine), which is highly localized in the olfactory bulb and exhibits antioxidant properties [27,28]. This highlights the vital importance of unveiling additional associations between these multifunctional metabolites and circadian brain function in order to obtain novel insights into sleep-specific neurochemical adaptations.

Mass spectrometry (MS)-based metabolomics constitutes a valuable tool for the comprehensive analysis of amino acids and analogues in brain tissue [29]. In addition, the metabolic profiling and mapping of several brain regions has proven to be a most suitable approach for detecting detailed and area-specific metabolites, since the brain tissue is highly inhomogeneous [27,30,31,32]. By applying this methodology, we have previously demonstrated region-specific metabolic alterations induced by sleep in the murine brain, including oxidative stress, energy metabolism and gut microbiota-derived molecules [7]. In the present study, we have now investigated an extended and specific profile of amino acid analogues to obtain a comprehensive sleep-specific metabolic profiling of the mouse CNS. Our findings demonstrate a series of region-specific changes of amino acid analogue levels, including *N*-acyl glycine, glutamate, dipeptides, and *N*-acetylated products in the context of the sleep/wake cycle, for the first time.

## 2. Results

### 2.1. Regional Brain Metabolomics of Amino Acid Analogues

**Figure 1:**
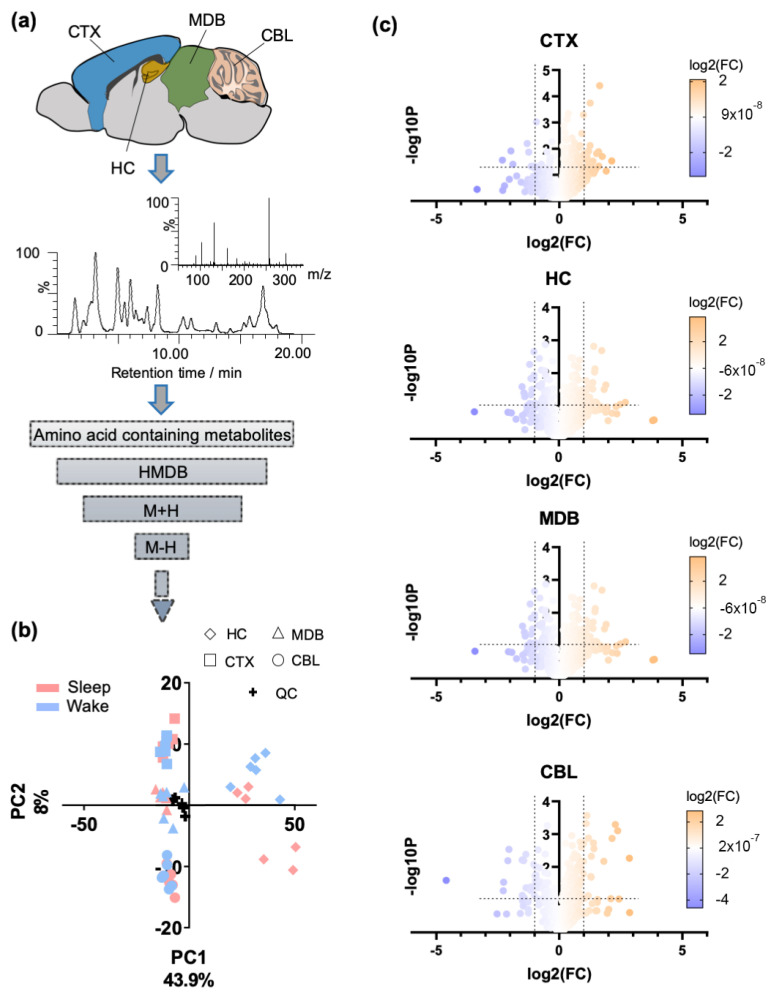
Overview of the metabolomics analysis of brain regions selective for amino acids and modifications. (**a**) UPLC-MS/MS-based metabolomics was applied for the analysis of amino acid analogues in four distinct brain regions in positive and negative ionization mode. The investigated brain regions are illustrated in a sagittal mouse brain tissue section. Brain tissue samples were collected from animals in sleep and wake state (N = 6 per group). (**b**) Principal component analysis on the different mouse brain regions is based on amino acid-containing metabolites (features) (N = 6 per group). (**c**) Volcano plots of the included features per brain region in both positive and negative ionization mode (two-tailed unpaired *t*-test on sleep/wake effects; *p* < 0.05). Significance is expressed as the negative log10 of the *p* value. Thresholds are defined as -log10*P* > 1.30 and log2(FC) > 1. The color scale represents the log2 fold change (FC), where FC = (sleep)/(wake); CBL: cerebellum; CTX: cortex; HC: hippocampus; MDB: midbrain.

Four distinct brain regions, i.e., the cortex (CTX), the hippocampus (HC), the midbrain (MDB), and the cerebellum (CBL), were collected from two groups of mice. The mice were sacrificed either during their subjective night (referred to as sleep state/10 a.m.) or during their subjective day (referred to as wake state/10 p.m.), respectively (N = 6 per group). The samples were extracted using our standard protocols and analyzed with UPLC-MS/MS-based metabolomics [7]. Isotopically labelled amino acids (phenylalanine, tyrosine and valine) and quality control (QC) samples were used to monitor the experimental procedure. The UPLC-MS data were processed with R using the XCMS metabolomics framework [33,34]. According to the HMDB-based tentative annotation of the extracted features, a total number of 749 amino acid-containing features identified in either positive or negative ionization mode were selected for further investigation and structure validation (Figure 1a).

Subsequently, the data distribution was evaluated using unsupervised principal component analysis (PCA) (Figure 1b). The QC samples were centered, and according to the first component (PC1), the highest variation was observed for the hippocampal (HC) samples. A small separation was detected between the two circadian states, mainly in the HC samples; we have thus investigated the effects of the sleep/wake cycle for these amino acid analogues individually for each brain region.

A number of significantly altered features between sleep and arousal states were detected (two-tailed unpaired *t*-test on sleep/wake effects, *p* < 0.05, Figure 1c). As illustrated in the volcano plots, sleep induced elevation and decline of particular features.

The circadian effects were studied for each individual brain region and extensive structural validation of the significant metabolites was performed. Metabolites were either validated via authentic standards (level 1 of confidence level) or via MS/MS fragmentation characterization (levels 2 and 3 of confidence level; Table S1) [35,36,37].

### 2.2. Sleep-Specific Effects on Acylated Amino Acids and Dipeptides in the Cerebral Cortex

**Figure 2:**
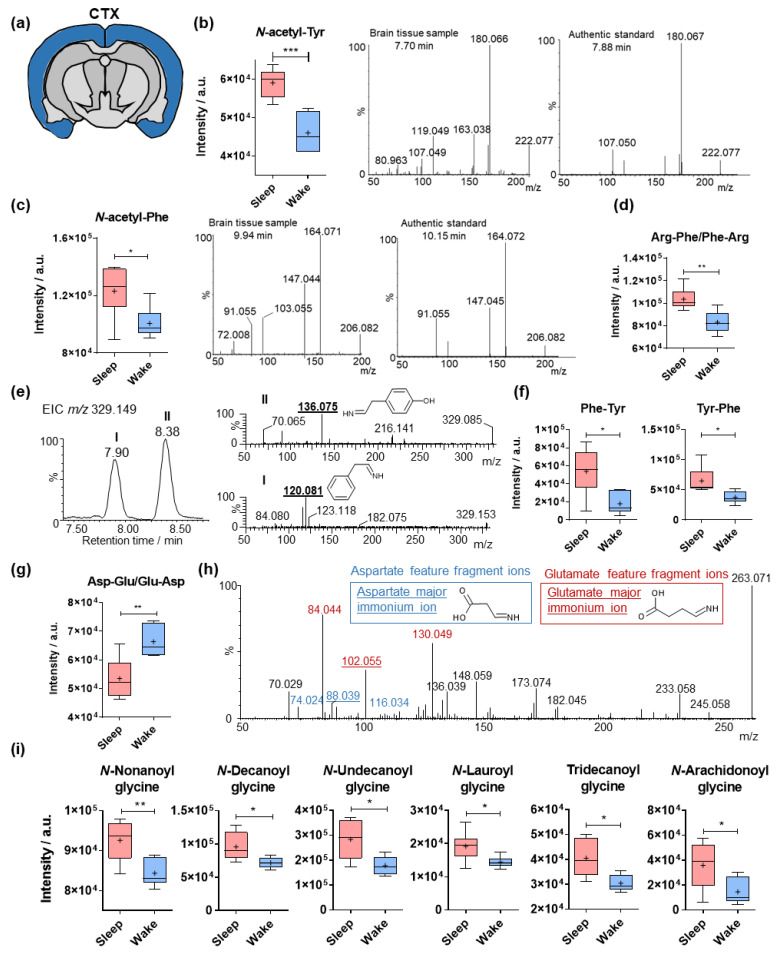
Sleep-specific alterations in the cortical levels of amino acid analogues. (**a**) Illustration of cerebral cortex (CTX) in a coronal mouse tissue section. (**b**) Mass spectrometric intensities of *N*-acetyl-tyrosine in the cerebral cortex (N = 6). Structure validation of *N*-acetyl-tyrosine was performed by comparison of tandem spectra collected from pooled brain sample and an authentic standard. (**c**) Mass spectrometric intensities of *N*-acetyl-phenylalanine in the cerebral cortex (N = 6). Structure validation of *N*-acetyl-phenylalanine was performed by comparison of tandem spectra collected from pooled brain samples and an authentic standard. (**d**) Mass spectrometric intensities of arginine- and phenylalanine-containing dipeptide in the cerebral cortex (N = 6). (**e**) Extracted ion chromatogram of *m*/*z* 329.149 in positive ionization mode and structural validation by tandem spectra collected from pooled brain sample. The 2 different chromatographic peaks at 7.90 and 8.38 min are annotated as **I** and **II**, respectively. The immonium product ions from the protonated amino acids are underlined and structurally illustrated. (**f**) Mass spectrometric intensities of tyrosine- and phenylalanine-containing dipeptides in the cerebral cortex (N = 6). (**g**) Mass spectrometric intensities of aspartate- and glutamate-containing dipeptide in the cerebral cortex (N = 6). (**h**) Structural validation of the aspartate- and glutamate-containing dipeptide by tandem spectra collected from pooled brain sample. (**i**) Mass spectrometric intensities of multiple *N*-acyl-glycines in the cerebral cortex (N = 6). Error bars: min and max of the median; plus signs represent the mean values; two-tailed unpaired *t*-test: * *p* < 0.05, ** *p* < 0.01, *** *p* < 0.001.

The cerebral cortex (CTX) occupies a large part of the cerebrum and is involved in principal executive functions (Figure 2a). The *N*-acetylated analogues of the aromatic amino acids tyrosine (*N*-AcTyr) and phenylalanine (*N*-AcPhe) were significantly elevated during sleep in the investigated cortical samples (Figure 2b,c). Isotopically labeled standards are required for each metabolite to determine the absolute concentration [20,38,39]. The structure of these two metabolites was validated via MS/MS spectra in comparison with authentic standards in negative ionization mode (Figure 2b,c). The small differences observed in retention time (<0.3 min) can be attributed to LC-MS/MS measurements of different matrices.

Several dipeptides containing the unacetylated aromatic amino acids were also detected to be significantly upregulated during sleep (Figure 2d–f). The structural validation of these molecules was primarily performed via MS/MS in positive ionization mode based on the feature product ions derived for each protonated amino acid [35]. Moreover, the acquired product ion spectra were compared with MS/MS spectra of the corresponding authentic standards available in the GNPS database [40] (Figures S1 and S2). The immonium product ion at *m/z* 120.080 is indicative of the presence of Phe in the molecule, and its relative intensity designates the C-terminal of the dipeptide, as shown in the examples for Arg-Phe and Phe-Arg (Figure S1). The isobaric dipeptides at *m*/*z* 329.149 eluting at different retention times were also structurally elucidated via the specific immonium product ions at *m*/*z* 120.080 and 136.075 for Phe and Tyr, respectively (Figure 2e and Figure S2). The dipeptide containing glutamate (Glu) and aspartate (Asp) was highly elevated during wakefulness (Figure 2g). Tandem MS analysis revealed the expected feature productions of Glu and Asp, including the characteristic immonium ions of their respective protonated species (Figure 2h).

A series of fatty acid acylated glycine (Gly) analogues were found to be significantly elevated during sleep (Figure 2i and Figure S3). Conjugated fatty acids were mainly saturated except for *N*-arachidonoyl Gly. This is also reflected by the correlation between the retention time and the *m*/*z* value, the linear range of which is obtained for the saturated analogues, while a polynomial relationship was observed for all acylated glycines (Figure S3).

### 2.3. Circadian Effects on Different Amino Acid Analogues in the Hippocampus, Midbrain and Cerebellum

The aromatic amino acids Phe, Tyr and tryptophan (Trp) have crucial roles in CNS metabolism and neurotransmitter synthesis. Phe and Tyr serve as precursors in the synthesis of catecholamines (i.e., dopamine and norepinephrine), while Trp is the precursor of serotonin (5-hydroxytryptamine). Analogues of these amino acids were found to be significantly affected by sleep in the hippocampus (HC), the midbrain (MDB) and the cerebellum (CBL).

**Figure 3:**
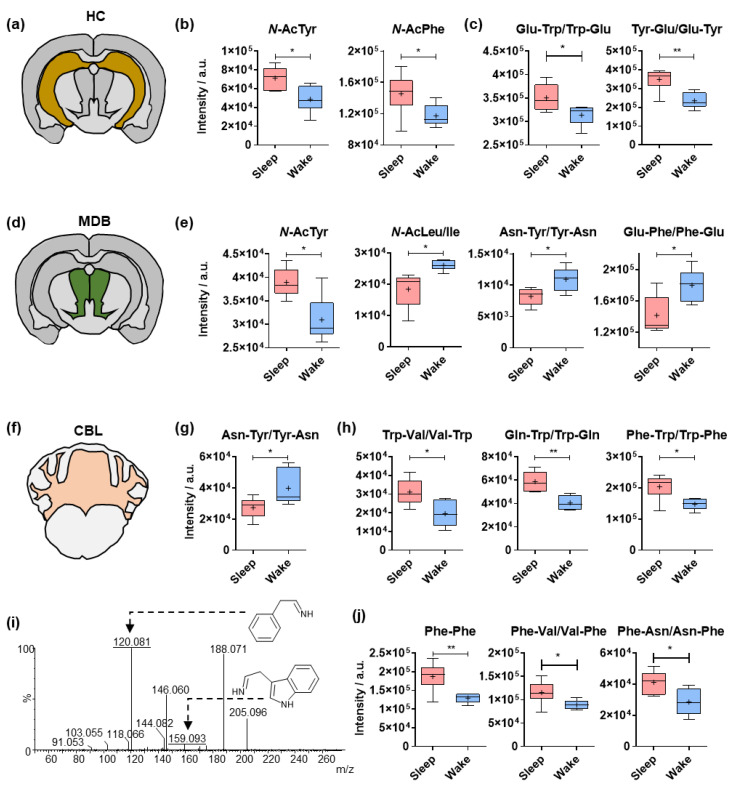
Sleep-specific alterations in the brain regional levels of amino acid analogues. (**a**) Illustration of the hippocampus (HC) in a coronal mouse tissue section. (**b**) Mass spectrometric intensities of *N*-acetyl-tyrosine and *N*-acetyl-phenylalanine in the hippocampus (N = 6). (**c**) Mass spectrometric intensities of representative tryptophan (Trp)- and tyrosine (Tyr)-containing dipeptides in the hippocampus (N = 6). (**d**) Illustration of the midbrain (MDB) in a coronal mouse tissue section. (**e**) Mass spectrometric intensities of representative amino acid analogues in the midbrain (N = 6). (**f**) Illustration of the cerebellum (CBL) in a coronal mouse tissue section. (**g**) Mass spectrometric intensities of representative tyrosine (Tyr)-containing dipeptides in the cerebellum (N = 6). (**h**) Mass spectrometric intensities of representative tryptophan (Trp)-containing dipeptides in the cerebellum (N = 6). (**i**) Structure validation of phenylalanine (Phe)- and tryptophan (Trp)-containing dipeptide by tandem spectra collected from pooled brain sample. The immonium product ions from the protonated amino acids are underlined and structurally illustrated. (**j**) Mass spectrometric intensities of representative phenylalanine (Phe)-containing dipeptides in the cerebellum (N = 6). Error bars: min and max of the median; plus signs represent the mean value; two-tailed unpaired *t*-test: * *p* < 0.05, ** *p* < 0.01.

HC is a subcortical structure of the cerebrum and a component of the limbic system which has a high involvement in learning and memory (Figure 3a). Acetylated Tyr and Phe were elevated during sleep, mirroring the results for the CTX (Figure 3b). Trp and Tyr containing dipeptides with Glu were significantly affected by the sleep/wake cycle in the HC (Figure 3c).

MDB is located in the brainstem, which is a region containing catecholaminergic, cholinergic and serotonergic nuclei (Figure 3d). Modified amino acids such as acetyl-Tyr and *N*-AcLeu/Ile have demonstrated sleep-induced changes, as have Tyr- and Phe-containing dipeptides (Figure 3e).

The most posterior part of the mammalian brain anatomically connected to the MDB is the CBL (Figure 3f). Multiple significant circadian-related alterations were detected in this brain region. Similar to the MDB, Tyr conjugated to asparagine (Asn) was significantly higher during wakefulness (Figure 3g). On the contrary, several Trp-containing dipeptides were found to be upregulated during sleep (Figure 3h). The structural validation of these dipeptides was also based on the feature fragments of the protonated amino acids (Figure 3i). The same effect was also observed for several Phe-containing dipeptides, which demonstrated significantly higher cerebellar levels in the sleep group (Figure 3j).

### 2.4. Comparative Analysis of Dipeptides between the Investigated Brain Regions

**Figure 4:**
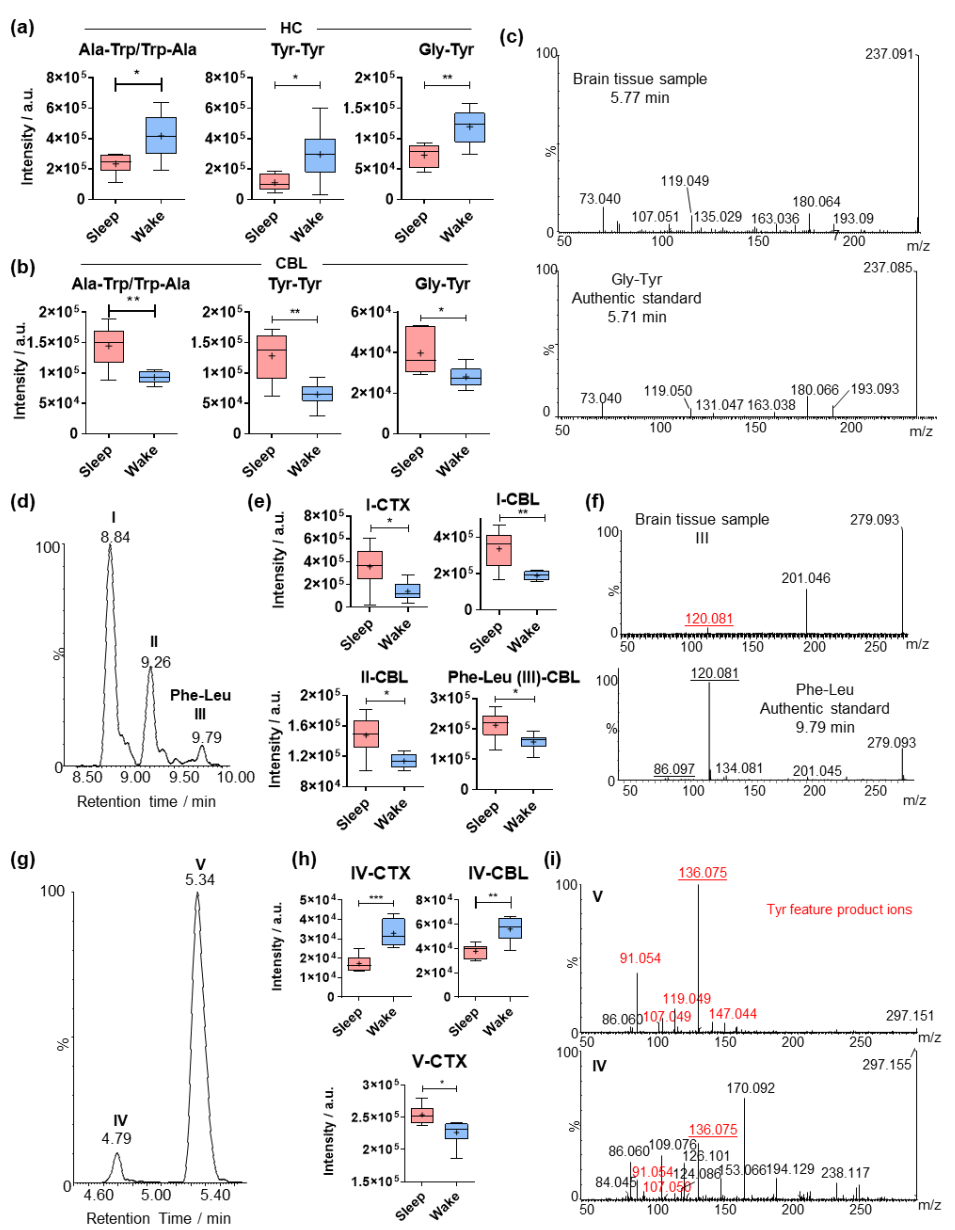
Comparison of significantly altered dipeptides in the investigated brain regions. (**a**) Mass spectrometric intensities of representative tyrosine (Tyr)-containing dipeptides in the hippocampus (N = 6). (**e**) Structure validation of glycyl-tyrosine (Gly-Tyr) by comparison of tandem spectra collected from pooled brain sample and an authentic standard. Extracted ion chromatogram of *m*/*z* 279.170 in positive ionization mode. The 3 different chromatographic peaks at 8.84, 9.26 and 9.79 min are annotated as **I**, **II** and **III**, respectively; (**b**) Mass spectrometric intensities of the 3 isobaric dipeptides (corresponding to the chromatographic peaks for **I**, **II** and **III**) in the cortex and the cerebellum (N = 6). (**c**) Structure validation of the dipeptides **I**, **II** and **III** by MS/MS tandem fragmentation spectra collected from pooled brain sample. (**d**) Extracted ion chromatogram of *m*/*z* 295.165 in positive ionization mode. The 4 different chromatographic peaks at 7.18, 7.33, 7.59, and 7.94 min are annotated as **IV**, **V, VI** and **VII**, respectively. (**e**) Mass spectrometric intensities of the dipeptide **V** in the cerebellum (N = 6). (**f**) Structure validation of the dipeptides **IV**, **V, VI** and **VII** by tandem spectra collected from pooled brain sample. The immonium product ions from the protonated amino acids are underlined. (**g**) Extracted ion chromatogram of *m*/*z* 297.107 in positive ionization mode. The 2 different chromatographic peaks at 4.79 and 5.34 min are annotated as **VIII** and **IX,** respectively. (**h**) Mass spectrometric intensities of the 2 isobaric dipeptides (corresponding the chromatographic peaks **VIII** and **IX**) in the cortex and cerebellum (N = 6). (**i**) Structure validation of the dipeptides **VIII** and **IX** by tandem spectra collected from pooled brain sample. Error bars: min and max of the median; plus signs represent the mean value; two-tailed unpaired *t*-test: * *p* < 0.05, ** *p* < 0.01, *** *p* < 0.001.

For a number of Trp- and Tyr-containing dipeptides, sleep induced the opposite effect in the HC compared with the CBL (Figure 4a,b). The dipeptides Ala-Trp/Trp-Ala, Tyr-Tyr and Gly-Tyr were significantly elevated during wakefulness in the HC (Figure 4a). On the contrary, the same dipeptides demonstrated higher cerebellar levels during sleep (Figure 4b), indicating region-specific functionalities. The use of the glycyl Tyr (Gly-Tyr) authentic standard allowed for the validation of the chemical structure and the determination of the C-terminus of the Gly-Tyr dipeptide (Figure 4c).

In many cases, multiple chromatographic peaks were extracted for a specific *m*/*z* value demonstrating region specific circadian effects (Figure 4d–g). The most representative examples were the two aromatic amino acids Phe and Tyr conjugated to either leucine or isoleucine (Leu/Ile/Figure 4d–f and Figure S5), which have been reported to display anxiolytic properties [12]. A number of dipeptides with Leu/Ile and Phe were found to be elevated during sleep in CTX and CBL (Figure 4a,b). The authentic standard of Phe-Leu was used for the identification of metabolite III based on rt and tandem MS/MS (Figure 4f). In the case of Tyr-containing dipeptides, only metabolite V was significantly increased in the CBL of the sleep group (Figure S5). As it is not possible to distinguish between Leu and Ile, comparison of the product ion spectra acquired in a pooled brain sample was performed with available spectra from the GNPS libraries suggests that metabolite **V** is Leu/Ile-Tyr (Figure S6). Interestingly, diverse circadian effects were observed for Asp and Tyr-containing dipeptides, depending on the C-terminal and the brain region (Figure 4g–i). Specifically, the first eluting dipeptide was elevated during wakefulness in CTX and CBL, while the second one was higher in the CTX tissue in the sleep group. We provide an overview of fold-changes and *p*-values for the identified metabolic differences in Figure S7 and Table S1.

## 3. Discussion

Amino acid modifications and dipeptides constitute a large group of metabolites with a wide range of biological properties. In the present study, we have examined the circadian related alterations of modified amino acids and dipeptides in four different mouse brain regions. Our approach has provided valuable new insights into amino acid modifications associated with the sleep/wake cycle while simultaneously offering an “amino acid analogue mapping” of the mammalian brain.

*N*-Acyl amino acids, including acetylated Tyr and Phe (CTX, HC, MDB) and Gly acylated with medium- to long-chain fatty acids (CTX) were found to be significantly elevated during sleep. *N*-Acyl amino acids have been reported as important endogenous signaling molecules [14]. *N*-Acyl glycines are present in the CNS, and the *N*-arachidonoyl analogue has been mostly studied. A wide spectrum of neuro-physiological properties was previously attributed to this metabolite, including analgesia and neuroprotection [14,26]. It was found that the analgesic activity of *N*-arachidonoyl Gly is mediated through modulation of glycinergic neurotransmission [41]. We have previously reported sleep-induced elevation of acyl-carnitines, demonstrating the relevance of lipid conjugation of small metabolites in brain regional circadian alterations [7]. Indeed, *N*-arachidonoyl Gly and oleoyl-carnitine have demonstrated similar mechanisms of exerting palliative activity through binding to the glycine transporter GlyT2 [42]. Our findings, thus, reveal new perspectives regarding the beneficial role of sleep for brain function by the regional increase of signaling molecules.

Sleep-induced alterations were detected for multiple dipeptides that mainly contain the three aromatic amino acids Phe, Tyr and Trp, as well as non-aromatic amino acids Glu and Asp. The connection between sleep and memory consolidation has been well established [2,43]. In particular, dipeptides containing Phe, Tyr or Trp have a wide range of neuroactive properties connected to memory enhancement [18,44,45]. These amino acids also play a key role in catecholamine synthesis and are vital for CNS function. Moreover, Leu conjugates of Phe and Tyr are known to have anxiolytic properties [12]. Thus, it is of high importance to investigate sleep-specific dipeptide alterations that were previously associated with cognitive augmentation. Herein, we have detected elevated cortical and cerebellar levels of dipeptides containing Leu/Ile with either Phe or Tyr during sleep that could indicate a potential connection between sleep and stress regulation.

Most of the Asp and Glu conjugates were elevated during wakefulness. Glu and Asp are excitatory neurotransmitters and therefore expected to be involved in neuronal activity performed in wakefulness. Interestingly, opposite effects were observed for several dipeptides in the HC compared to other brain regions. In particular, Tyr-containing dipeptides were significantly higher in the HC of the wake group compared to the sleep group, while the same molecules were identified to be elevated during sleep in the cerebellum. These metabolic differences reflect the distinct functionalities of these regions during sleep. HC is the major brain region involved in memory and cognitive function, while CBL is involved in motor control. However, a recent study proposed a novel neural network of cerebello-hippocampal interactions, potentially suggesting complementary metabolic needs [46]. This finding also emphasizes the importance of the brain’s regional metabolomic analysis, as recent mapping of the regional distribution of dipeptides revealed particular localization patterns [47,48]. In addition, differential tissue distribution has been observed for isomeric dipeptides depending on the C-terminal amino acid [49].

## 4. Materials and Methods

### 4.1. Chemicals

Solvents and reagents were purchased from SigmaAldrich (Merck, Germany) or Fisher Scientific (Hampton, NH, USA) and were used without further purification. Phe-Leu was provided by Biosynth Carbosynth (Compton, UK). The in-house built metabolite library was obtained from MetaSci (Toronto, ON, Canada). Mass spectrometry-grade solvents were used for UPLC-ESI-MS analysis.

### 4.2. Animal Experiments

The animal study was performed as previously described [7]. Briefly, brain tissue was rapidly extracted (<2 min) from adult male C57BL/6 mice (Janvier Labs) after euthanasia by cervical dislocation at either 10 a.m. or 10 p.m. All experiments were performed according to ethical approval from the Malmö-Lund Ethical Committee on Animal Research (Dnr 5.8.18- 08269/2019) and conducted according to the CODEX guidelines by the Swedish Research Council, Directive 2010/63/EU of the European Parliament on the protection of animals used for scientific purposes and Regulation (EU) 2019/1010 on the alignment of reporting obligations. This study complies with the ARRIVE guidelines [50]. Brains were dissected to isolate the cortex, hippocampus, midbrain and cerebellum. Brain regions were then placed in Eppendorf tubes and snap frozen at −80 °C in liquid nitrogen.

### 4.3. Tissue Processing and Sample Preparation

Every brain region sample was weighed and transferred into bead-containing vials where methanol:water (80:20) was added (approx. 4 mL/mg brain tissue). As an internal standard (I.S.), a mixture of C-13 isotopically labeled tyrosine (5 mg/mL), phenylalanine (10 mg/mL) and valine (30 mg/mL) was used [7]. The homogenization was performed in a Lysing matrix D instrumentation (MP Biomedicals) in dry ice at a cycle of 20 s shaking (4 m/s) and 30 s performed 3 times. Samples were collected, precipitated on ice for 1 h and centrifuged at 13,400 rpm for 5 min. The supernatant was collected and dried under vacuum on a Speedvac and subsequently stored in −20 °C for a maximum of 3 days prior to analysis. Samples were re-suspended with water:acetonitrile (95:5) prior to UPLC-MS/MS analysis at a volume normalized to the sample weight. Quality control (QC) samples were prepared by 5 μL aliquots from all samples.

### 4.4. UPLC Mass Spectrometry

The UPLC-MS/MS analysis was performed in a SYNAPT G2-S high-definition mass spectrometer (HDMS) using an electrospray ionization (ESI) source with an AQCUITY UPLC I-class system and equipped with a Waters ACQUITY UPLCVR HSS T3 column (1.8 mm, 100 2.1 mm) [7,20,51]. Water with 0.1% formic acid was used as mobile phase A and methanol with 0.1% formic acid was used as mobile phase B. The column temperature was kept at 40 °C, and the autosampler was kept at 6 °C. The flow rate was set to 0.2 mL/min. The gradient used was as follows: 0–2 min, 0% B; 2–15 min, 0–100% B; 15–16 min, 100% B; 16–17 min, 100-0% B; 17–21 min, 0% B. The system was controlled using the MassLynx software package v 4.1 from Waters. High-resolution mass spectra were acquired in positive and negative ionization mode at a mass range of *m*/*z* 50–1200. Data acquisition was performed in MSE mode. The samples were injected to the UPLC-MS system in a randomized order with QC samples injected in the beginning and end of the sample list in both ionization modes, as well as after every eight samples (7 QCs in each ionization mode in total). Tandem MS spectra were also collected with a Maxis II ETD Q-TOF mass spectrometer equipped with an Elute UHPLC system using the same chromatographic parameters.

### 4.5. Identification of Metabolites

Significant features were primarily annotated by the human metabolome data base (www.hmdb.ca, accessed on 21 December 2021) based on their *m*/*z* value and given the high mass accuracy provided by the mass analyzer [52]. Amino acid analogues included in the in-house built standard library and standards of dipeptides were measured in the same UPLC-MS/MS system and used for the assignment of the retention time (rt). Tandem MS experiments were performed in brain tissue samples in positive or negative ionization mode with CID of 20 eV, depending on the analyte. The product ion spectra were compared to the corresponding standards or to MS/MS spectra of authentic standards available in the GNPS libraries and acquired in similar instrumental systems, i.e., ESI-QTOF [40,53].

### 4.6. Data Analysis

The chromatograms and mass spectra were processed using the XCMS R package (version 1.4.414) for peak alignment and retention time correction [33,34] in both positive and negative ionization mode. From the corresponding feature lists obtained from the software, features with intensities > 20,000 ion count, rt > 1 min and %CV of the QCs < 30 were selected for further statistical analysis as considerably higher than noise. Subsequently, according to the HMDB-based putative annotation of the extracted features, a total number of 749 amino acid-containing features in both positive and negative ionization mode measured in 4 different brain regions (CBL, CTX, HC and MDB) of both groups (sleep-wake, N = 6 per group) were selected. The intensities of the included internal standards and the QC samples were plotted against the UPLC-MS/MS sample injection order to evaluate the stability and performance of the experimental set over time. An overview of the data was provided by principal component analysis (PCA), prior to which the data was autoscaled using the metabolomics platform Metaboanalyst [54]. The normality of the test statistics and *p* values were evaluated using the same platform, and the data were distributed normally. For the hypothesis testing, two-tailed *t*-tests were applied in metabolites extracted from every region (CBL, CTX, HC, MDB) for detecting consciousness state (sleep/wake) differences.

## 5. Conclusions

The UPLC-MS/MS methodology that was applied in our study enabled the detection and mapping of multiple amino acid analogues with high accuracy. Validation of the metabolite structure using tandem MS/MS analysis led to the identification of the most informative product ion spectra validating the presence of significantly altered amino acids. We also invested in the identification of the correct structure of many C-terminal dipeptides for significantly altered modified dipeptides using standards or MS/MS libraries. This approach provided a high level of confidence for the metabolite structures. Authentic standards are required to completely validate the structures of all isobaric and isomeric dipeptides and distinguish between D- and L-amino acids in future studies.

In summary, our study provides, for the first time, a comprehensive analysis of the circadian impact on the levels of amino acids and their analogues in distinct brain regions. Our findings highlight the complexity of the connection between the sleep/wake cycle and the brain metabolome. We believe that these findings should be included in future animal models for the investigation of sleep disorders, such as fatal familial insomnia and sleep deprivation related to neurodegenerative diseases. Dipeptides have also demonstrated neurophysiological properties when administered exogenously [55]. Therefore, our findings can serve as a surrogate for the design and development of sleep-induced or circadian-related agents. In addition, from the high conservation of the reported metabolites among species emerges the translational potential of our study to other mammals, including humans.

## Data Availability

The raw MS data has been uploaded to the metabolomics data repository Metabolights with the number MTBLS3811 (www.ebi.ac.uk/metabolights/MTBLS3811, accessed on 21 December 2021) [56].

## Supplementary Materials

The following are available online at https://www.mdpi.com/article/10.3390/metabo12010021/s1. Figure S1: Structural validation of the dipeptide Arg-Phe/Phe-Arg via tandem MS, Figure S2: Structural elucidation of the dipeptides Phe-Tyr and Tyr-Phe, Figure S3: Structural validation of a series of *N*-acyl-glycines, Figure S4: Structural elucidation of the dipeptides Phe-Trp and Trp-Phe, Figure S5: Comparison of multiple isobaric dipeptides, Figure S6: Structural elucidation of the dipeptides Ile-Tyr, Tyr-Ile and Tyr-Leu, Figure S7: Summary of the presented significant metabolites in all investigated brain regions, Table S1: Structure validation of significantly altered amino acid analogues.
