# Supplementary material for: Regional Brain Analysis of Modified Amino Acids and Dipeptides during the Sleep/Wake Cycle
Source: Metabolites. 2021 Dec 27;12(1):21. doi: 10.3390/metabo12010021 (PMC8780251; doi:10.3390/metabo12010021)
Supplement: Supplementary file 1 [file metabolites-12-00021-s001.zip › metabolites-1521903-supplementary.pdf]

Supplementary Materials for

Regional brain analysis of modified amino acids and dipeptides during the sleep/wake cycle

Theodosia Vallianatou [1], Nicholas B. Bèchet [2] Mario S.P. Correia[1], Iben Lundgaard[2,3] and Daniel Globisch[1,*]

Affiliations

[1] Department of Chemistry-BMC, Science for Life Laboratory, Uppsala University, Box 599, SE-75124 Uppsala, Sweden

[2] Department of Experimental Medical Science, Lund University, SE- 22362 Lund, Sweden

[3] Wallenberg Centre for Molecular Medicine, Lund University, SE-22362 Lund, Sweden

*Corresponding author. Email: Daniel.Globisch@scilifelab.uu.se

Table of Contents

Supplementary figures 3
Fig. S1. Structural validation of the dipeptide Arg-Phe/Phe-Arg via tandem MS. 3
Fig. S2. Structural elucidation of the dipeptides Phe-Tyr and Tyr-Phe. 4
Fig. S3. Structural validation of a series of *N*-acyl glycines 5
Fig. S4. Structural elucidation of the dipeptides Phe-Trp and Trp-Phe. 6
Fig. S5. Comparison of multiple isobaric dipeptides. 7
Fig. S6. Structural elucidation of the dipeptides Ile-Tyr, Tyr-Ile and Tyr-Leu. 8
Fig. S7. Summary of the presented significant metabolites in all investigated brain regions. 9
Supplementary tables 10
Table S1. Structure validation of significantly altered amino acid analogues 10
References 12

Supplementary figures

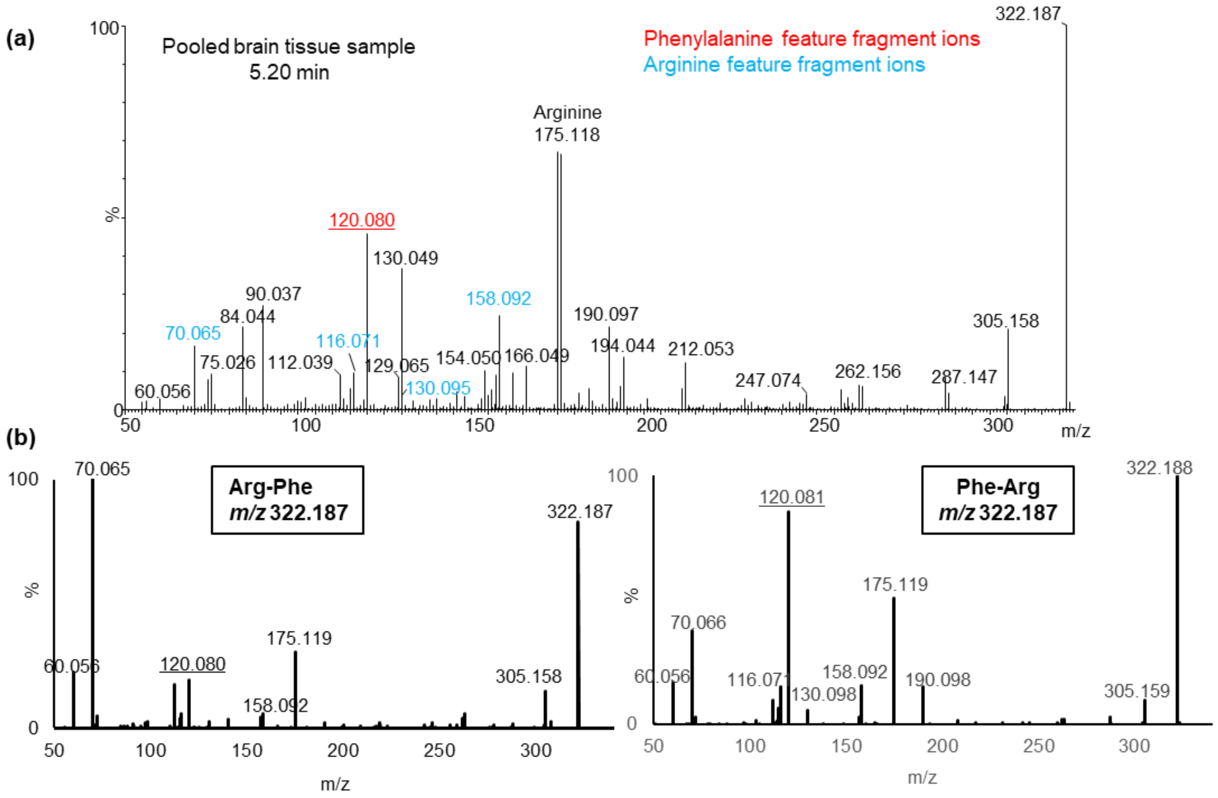

Fig. S1. Structural validation of the dipeptide Arg-Phe/Phe-Arg via tandem MS. (**a**) Pooled brain tissue was used for the UPLC-MS/MS analysis and spectra acquired at 20 eV. The feature product ions derived from the protonated amino acids are highlighted in red and blue for Phe and Arg, respectively (1). The immonium product ion of the protonated Phe is underlined; (**b**) Experimental MS/MS spectra available in the GNPS (2) database for Arg-Phe and Phe-Arg.

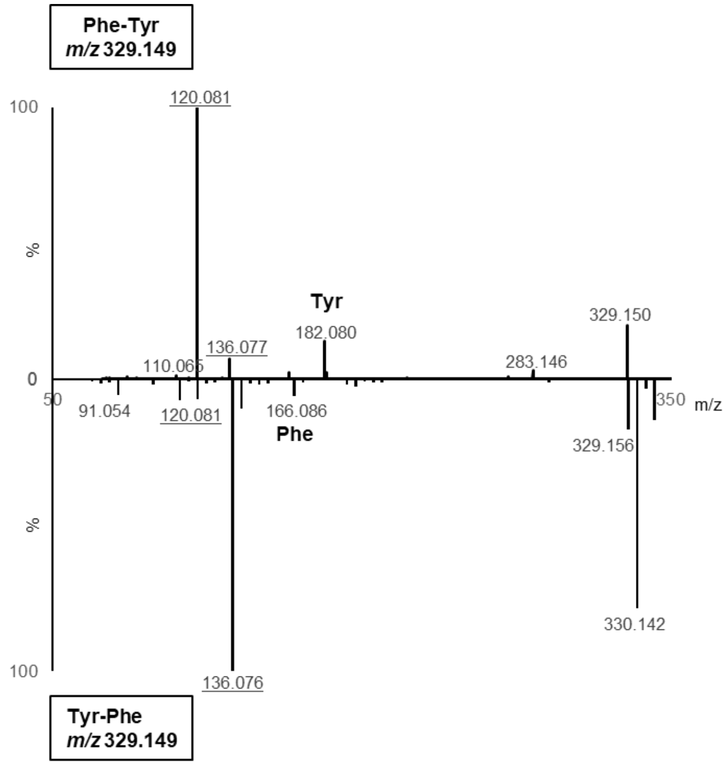

Fig. S2. Structural elucidation of the dipeptides Phe-Tyr and Tyr-Phe. MS/MS spectra available in the GNPS (2) database for Phe-Tyr and Tyr-Phe.

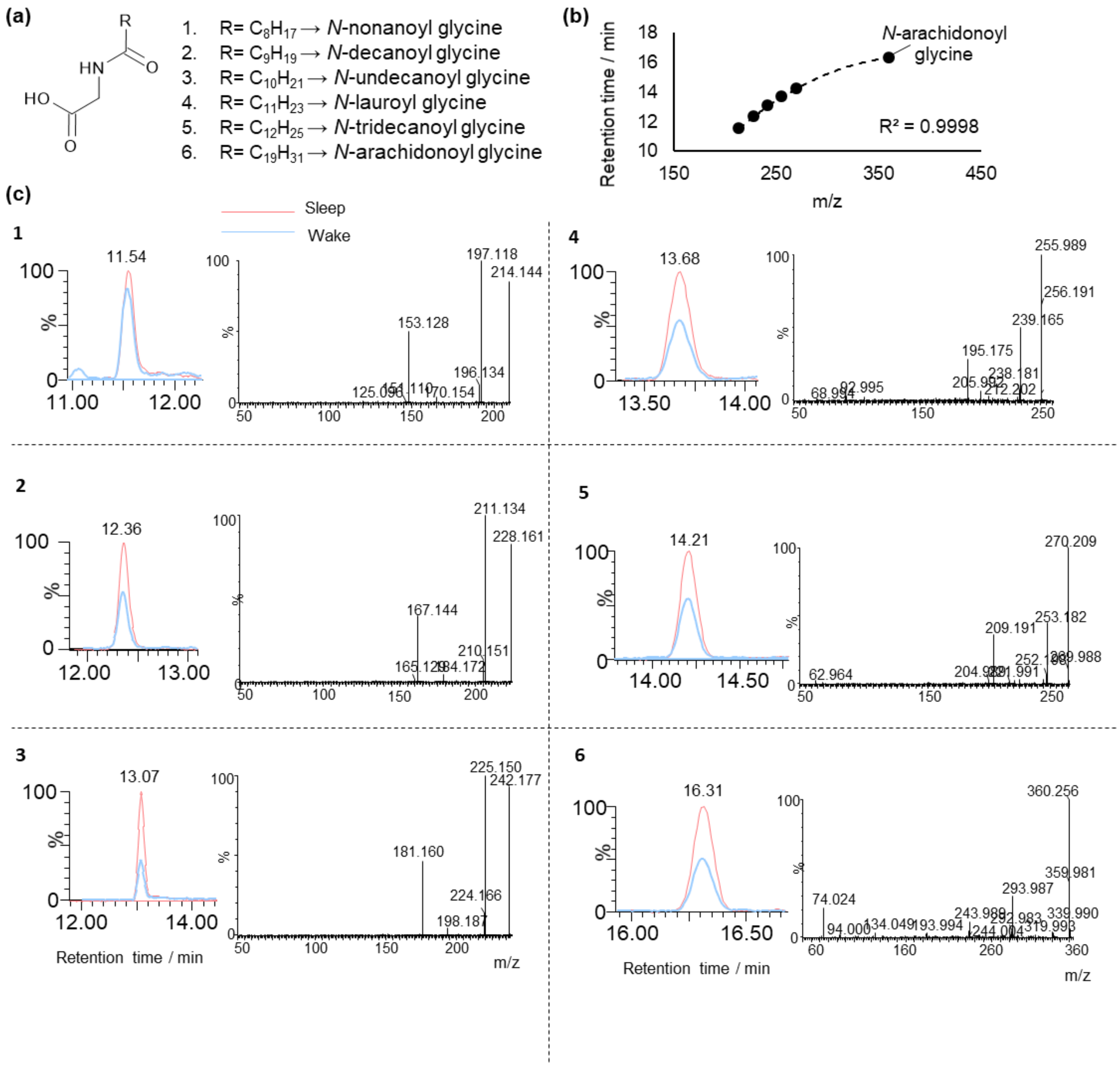

Fig. S3. Structural validation of a series of *N*-acyl glycines. **(a)** Chemical structure of N-acyl glycine analogues; **(b)** Second degree polynomial correlation between the m/z values of the significant *N*-acyl glycines and their retention time; **(c)** Extracted ion chromatograms and MS/MS spectra collected for the *N*-glycine analogues in brain tissue sample.

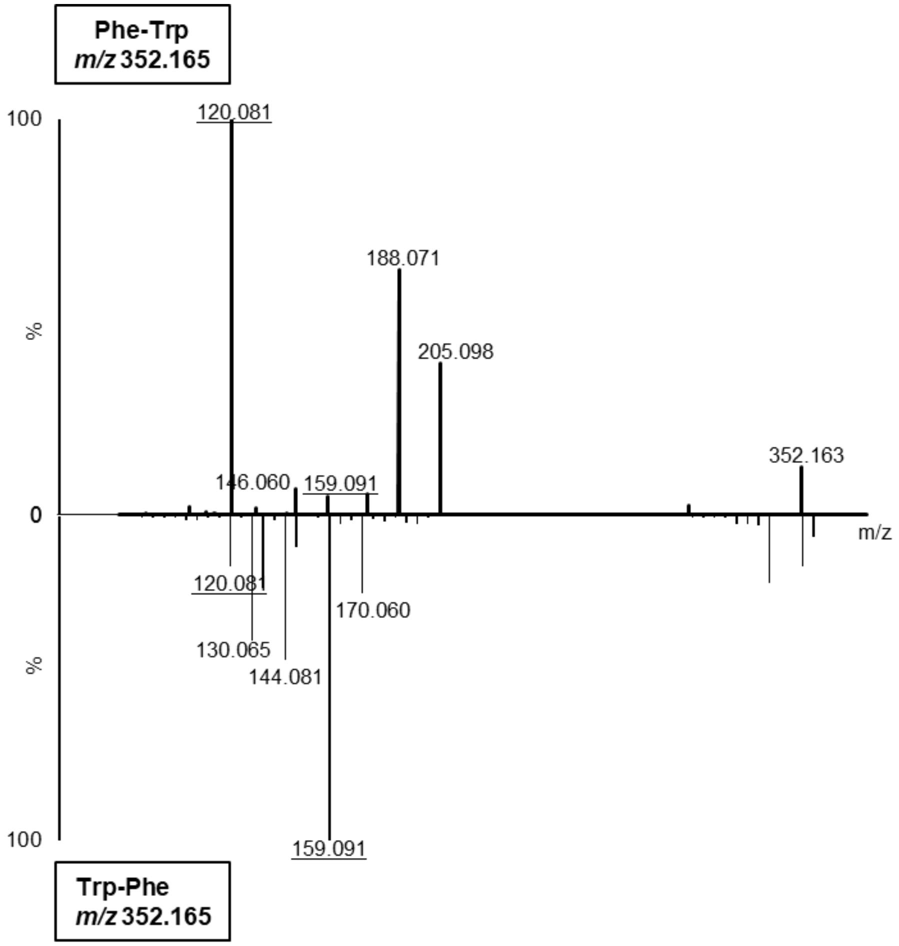

Fig. S4. Structural elucidation of the dipeptides Phe-Trp and Trp-Phe. MS/MS spectra available in the GNPS (2) database for Phe-Trp and Trp-Phe.

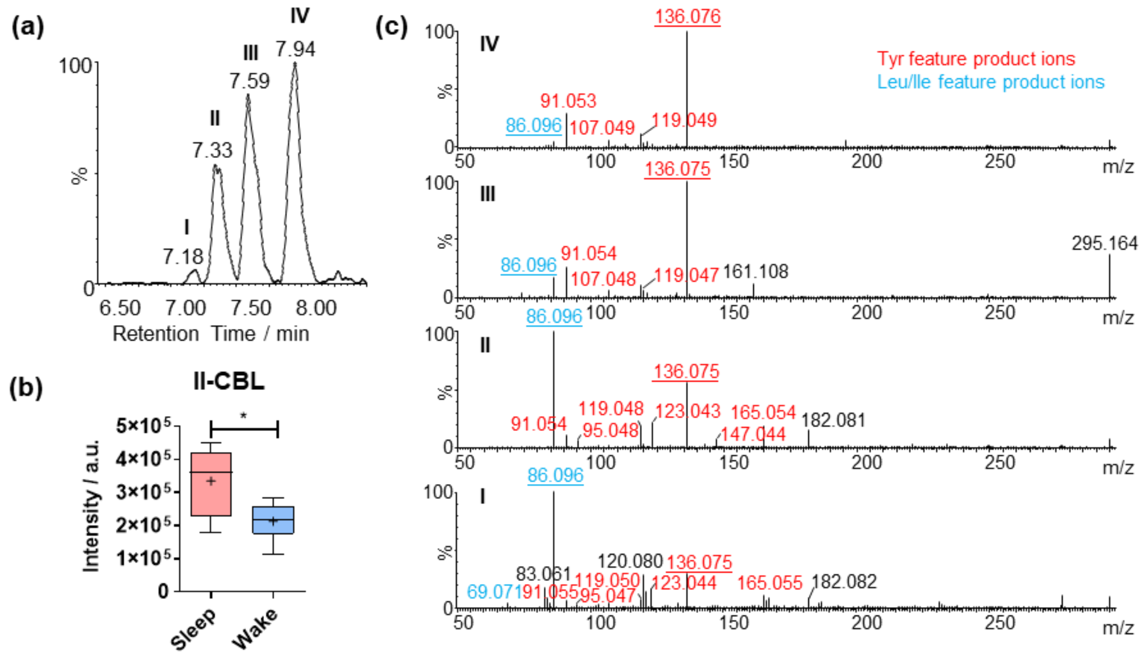

Fig. S5. Comparison of multiple isobaric dipeptides. (**a**) Extracted ion chromatogram of *m/z* 295.165 in positive ionization mode. The four different chromatographic peaks at 7.18, 7.33, 7.59 and 7.94 min are annotated as **I**, **II, III** and **IV**, respectively; (**b**) Mass spectrometric intensities of the dipeptide **II** in the cerebellum (N=6); (**c**) Structure validation of the dipeptides **I**, **II, III** and **IV** by tandem spectra collected from pooled brain sample. The immonium product ions from the protonated amino acids are underlined.

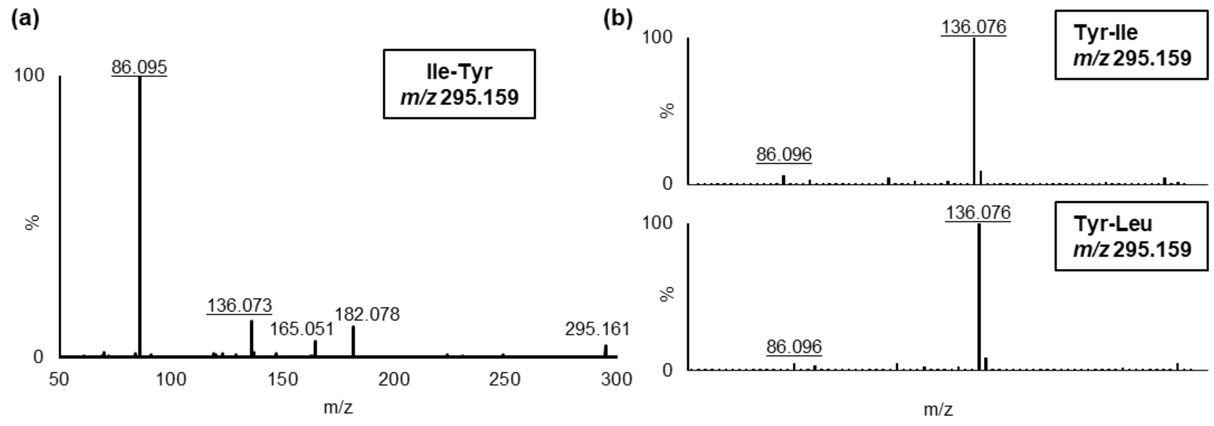

Fig. S6. Structural elucidation of the dipeptides Ile-Tyr, Tyr-Ile and Tyr-Leu. (a) MS/MS spectra available in the GNPS (2) database for Ile-Tyr; **(b)** MS/MS spectra available in the GNPS (2) database for Tyr-Ile and Tyr-Leu.

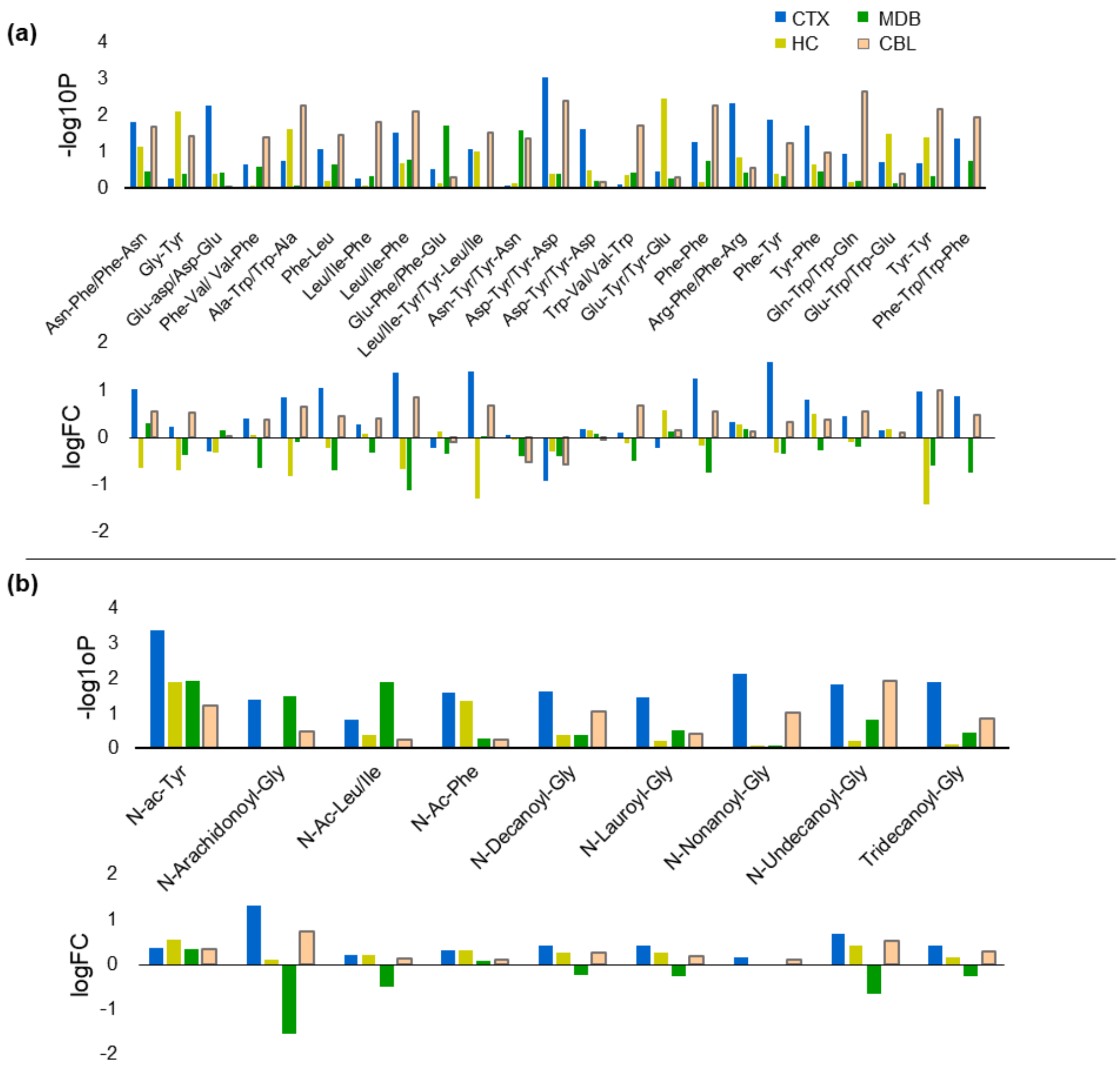

Fig. S7. Summary of the presented significant metabolites in all investigated brain regions. (**a**) Column plots of the significance, expressed as the negative log10 of the P value (-log10P > 1.30), and the log2 fold change (FC) of the presented dipeptides per brain region in both positive and negative ionization mode (two-tailed unpaired t-test on sleep/wake effects; P<0.05). (**b**) Column plots of the significance, expressed as the negative log10 of the P value (-log10P > 1.30), and the log2 fold change (FC) of the presented acylated amino acids per brain region in both positive and negative ionization mode (two-tailed unpaired t-test on sleep/wake effects; P<0.05); FC= (sleep)/(wake); CBL: cerebellum; CTX: cortex; HC: hippocampus; MDB: midbrain.

Supplementary tables

Table S1. Structure validation of significantly altered amino acid analogues

	m/z experimental	*m/z* theoretical	ppm	[a]Ionization mode	Rt brain tissue (min)	Rt authentic standard (min)	MS/MS	Confidence level*
N-Ac-Tyr	222.075	222.0761	4.95	NEG	7.55	7.81	20 eV	1
N-Arachidonoyl glycine	360.255	360.2533	-4.72	NEG	16.31		20 eV	2a
Asn-Phe/Phe-Asn	278.113	278.1135	1.80	NEG	6.21		20 eV	2a
Gly-Tyr	237.086	237.0870	4.22	NEG	5.63	5.85	20 eV	1
N-Ac-Leu/Ile	172.096	172.0968	4.65	NEG	8.19		20 eV	2b
N-Ac-Phe	206.080	206.0812	5.82	NEG	9.75	10.06	20 eV	1
N-Decanoylglycine	228.159	228.1594	1.75	NEG	12.35		20 eV	2a
N-Lauroylglycine	256.191	256.1907	-1.17	NEG	13.68		20 eV	2a
N-Nonanoylglycine	214.143	214.1438	3.74	NEG	11.54		20 eV	2a
N-Undecanoylglycine	242.175	242.1751	0.41	NEG	13.07		20 eV	2a
Tridecanoylglycine	270.207	270.2064	-2.22	NEG	14.21		20 eV	2a
Glu-Asp/Asp-Glu	263.086	263.0874	5.32	POS	1.85		20 eV	3
Phe-Val/ Val-Phe	265.155	265.1547	-1.13	POS	7.72		20 eV	3
Ala-Trp/Trp-Ala	276.133	276.1343	4.71	POS	7.10		20 eV	3
Phe-Leu	279.170	279.1703	1.07	POS	9.70	9.79	20 eV	1
Leu/Ile-Phe	279.170	279.1703	1.07	POS	9.18		20 eV	3

Leu/Ile-Phe	279.171	279.1703	-2.51	POS	8.77		20 eV	3
Glu-Phe/Phe-Glu	295.129	295.1288	-0.68	POS	8.15		20 eV	3
Leu/Ile-Tyr/Tyr-Leu/Ile	295.165	295.1652	0.68	POS	7.32		20 eV	3
Asn-Tyr/Tyr-Asn	296.124	296.1241	0.34	POS	6.15		20 eV	3
Asp-Tyr/Tyr-Asp	297.107	297.1081	3.70	POS	4.65		20 eV	2a
Asp-Tyr/Tyr-Asp	297.108	297.1081	0.34	POS	5.23		20 eV	2a
Trp-Val/Val-Trp	304.165	304.1656	1.97	POS	8.27		20 eV	3
Glu-Tyr/Tyr-Glu	311.124	311.1238	-0.64	POS	6.50		20 eV	3
Phe-Phe	313.155	313.1547	-0.96	POS	9.48		20 eV	2a
Arg-Phe/Phe-Arg	322.185	322.1874	7.45	POS	5.29		20 eV	2a
Phe-Tyr	329.149	329.1496	1.82	POS	7.90		20 eV	2a
Tyr-Phe	329.150	329.1496	-1.22	POS	8.39		20 eV	2a
Gln-Trp/Trp-Gln	333.156	333.1557	-0.90	POS	6.51		20 eV	3
Glu-Trp/Trp-Glu	334.139	334.1397	2.09	POS	6.91		20 eV	3
Tyr-Tyr	345.144	345.1445	1.45	POS	6.87		20 eV	2a
Phe-Trp/Trp-Phe	352.166	352.1656	-1.14	POS	9.42		20 eV	3

*Confidence level: Level 1- the proposed structure has been confirmed via appropriate measurement of an authentic reference standard with MS, MS/MS and retention time matching; Level 2a- matching literature or library tandem MS/MS spectrum data where the spectrum-structure match is unambiguous; Level 2b/ “Diagnostic” - represents the case where no other structure fits the experimental information, but no standard or literature information is available for confirmation; Level 3- evidence exists for possible structure(s), but insufficient information for one exact structure only (e.g., positional isomers). *(3, 4)*

References

1. Zhang P, Chan W, Ang IL, et al.: Revisiting Fragmentation Reactions of Protonated α-Amino Acids by High-Resolution Electrospray Ionization Tandem Mass Spectrometry with Collision-Induced Dissociation. Sci Rep 9: 6453, 2019.

2. Wang M, Carver JJ, Phelan VV, et al.: Sharing and community curation of mass spectrometry data with Global Natural Products Social Molecular Networking. Nat Biotechnol 34: 828-837, 2016.

3. Schymanski EL, Jeon J, Gulde R, et al.: Identifying small molecules via high resolution mass spectrometry: communicating confidence. Environ Sci Technol 48: 2097-2098, 2014.

4. Correia MSP, Jain A, Alotaibi W, Young Tie Yang P, Rodriguez-Mateos A and Globisch D: Comparative dietary sulfated metabolome analysis reveals unknown metabolic interactions of the gut microbiome and the human host. Free Radic Biol Med 160: 745-754, 2020.
